# Therapeutic Application of Synbiotics, a Fusion of Probiotics and Prebiotics, and Biogenics as a New Concept for Oral *Candida* Infections: A Mini Review

**DOI:** 10.3389/fmicb.2016.00010

**Published:** 2016-01-25

**Authors:** Tomoko Ohshima, Yukako Kojima, Chaminda J. Seneviratne, Nobuko Maeda

**Affiliations:** ^1^Department of Oral Microbiology, School of Dental Medicine, Tsurumi UniversityKanagawa, Japan; ^2^Department of Oral Sciences, Faculty of Dentistry, National University of SingaporeSingapore, Singapore

**Keywords:** probiotics, prebiotics, synbiotics, biogenics, oral candidiasis, lactobacilli

## Abstract

*Candida* is a major human fungal pathogen causing infectious conditions predominantly in the elderly and immunocompromised hosts. Although *Candida* resides as a member of the oral indigenous microbiota in symbiosis, some circumstances may cause microbial imbalance leading to dysbiosis and resultant oral candidiasis. Therefore, oral microbial symbiosis that suppresses the overgrowth of *Candida* is important for a healthy oral ecosystem. In this regard, probiotics, prebiotics, and synbiotics can be considered a potential therapeutic and preventive strategy against oral candidiasis. Prebiotics have a direct effect on microbial growth as they stimulate the growth of beneficial bacteria and suppress the growth of pathogens. Probiotics render a local protective effect against pathogens and a systemic indirect effect on immunological amelioration. Synbiotics are fusion products of prebiotics and probiotics. This mini review discusses the potential use and associated limitations of probiotics, prebiotics, and synbiotics for the prevention and treatment of oral candidiasis. We will also introduce biogenics, a recent concept derived from the work on probiotics. Biogenics advocates the use of beneficial bioactive substances produced by probiotic bacteria, whose activities are independent from the viability of probiotic bacteria in human bodies.

## Introduction

The indigenous microbiota on the surfaces of the skin and mucous membranes plays a role in preventing the invasion of foreign pathogenic microorganisms. The oral cavity possesses a diverse set of indigenous microbiota that perpetually interacts with the host mucosal surfaces. The oral microbiota predominantly comprises bacteria and a small proportion of fungi. *Candida* is the major fungus residing even in the healthy human oral cavity (Sardi et al., 2013). However, depending on circumstances, *Candida* can transform into a pathogen causing oral infections. Hence, when there is a collapse in the healthy microbial balance, i.e., dysbiosis, *Candida* can proliferate and cause a typical opportunistic infection. Oral candidiasis has been frequently observed in the elderly population due to problems associated with quality and the production of saliva, as well as decreased cell-mediated immunity (Scully et al., 1994). Systemic *Candida* infections such as *Candida* pneumonia and candidemia due to intravascular indwelling catheters have also been observed in elderly populations (Eggimann et al., 2003). Recurrent oral candidiasis occurs frequently in HIV-positive and AIDS patients (Scully et al., 1994). The administration of antifungal drugs is generally the first-line therapy of candidiasis. However, the emergence of drug-resistant strains and frequent recurrence of the disease in affected individuals are increasing challenges in antifungal therapy (Pfaller and Diekema, 2007). This has prompted the need for an alternative therapeutic and prevention strategy. In this mini review, we will succinctly discuss the potential use of probiotics, prebiotics, and synbiotics as an alternative antifungal therapy. In addition, a new concept of biogenics will be introduced. Biogenics is a strategy to overcome the potential disadvantage of synbiotics, including difficulties in the colonization process of non-native probiotic bacteria. It also provides an additional advantage to produce functional foods with bioactive metabolites.

## Probiotics

### The Definition and History

The term “probiotics,” in contrast to antibiotics, was proposed by Lilly and Stillwell (1965), from the original ecological term, “probiosis” used by Kollath (1953), meaning a symbiotic relationship between organisms. Fuller (1989) defined a probiotic as “A live microbial feed supplement which beneficially affects the host animal by improving its intestinal microbial balance” (Fuller, 1989). Hence, at that time, probiotics were intended to be used only for the “intestinal microbiota.” Subsequent studies revealed general health benefits of probiotics, such as an enhancement of the human immune system, preventive effects concerning urinary tract and respiratory tract infections and the allergic or atopic condition in infants (Gourbeyre et al., 2011). Hence, probiotics were redefined by Salminen et al. (1998) as “A viable microbial food supplement which beneficially influences the health of the host.” According to the FAO/WHO, probiotics are defined as “live microorganisms when administered in adequate amounts confer a health benefit on the host” (FAO/WHO, 2001).

### Clinical Trials of Probiotics for Oral *Candida* Infections

There has been a gradual increase in the number of studies that focus on the application of probiotics on oral health (Haukioja, 2010). The majority of these studies have focused on two major dental diseases, dental caries and periodontitis (Krasse et al., 2005; Vivekananda et al., 2010; Cagetti et al., 2013). However, studies on the use of probiotics for oral candidiasis are sparse (**Table 1**). Ahola et al. (2002) and Hatakka et al. (2007) conducted double-blinded, randomized clinical trials using probiotic cheese on elderly populations with some oral health problems and carriers of oral *Candida* compared with a younger cohort (18–35 years of age). There was an observed trend that the probiotics could decrease the quantity of *Candida*. However, the effect was not significant (Ahola et al., 2002) or was small without an improvement in the mucosal symptom (Hatakka et al., 2007). On the other hand, studies conducted by Mendonça et al. (2012), Ishikawa et al. (2015), and Kraft-Bodi et al. (2015) reported a slight or moderate improvement of oral candidiasis when patients were treated with probiotics. Dos Santos et al. (2009) reported a drastic improvement of oral candidiasis upon probiotic treatment.

**Table 1:** Summary of studies that examined the antifungal activity of probiotics against *Candida albicans.*

Reference	Test strains	Test design/Feature tested	Results
**Clinical studies**			
Ahola et al. (2002)	*L. rhamnosus GG/LS*	Intervention with cheese, Double-blinded placebo RCT	Reduction in the risk of a high level of *Candida*
Hatakka et al. (2007)	*L. lactis*, *L. helveticus*, *L. rhamnosus GG*, *P. freudenreichii*	Intervention of an elderly group with cheese for 16 weeks, Double-blinded randomized placebo trial (tested group, *n* = 136, control group, *n* = 140)	10% reduction of the high *Candida* count rate in the tested group (after 16-weeks intervention)
Dos Santos et al. (2009)	*L. casei.* *B. breve*	No control group, 26 individuals Intervention with a commercial probiotic drink for 20 days	Reduction of the *Candida* carrying rate, reduction of the sIgA level
Mendonça et al. (2012)	*L. casei*, *B. breve*	No control group, 42 individuals over 65 years of age Intervention with a commercial probiotic drink for 30 days	Decrement of *Candida* prevalence, increment of sIgA level
Sutula et al. (2013)	*L. casei*	No control group, 22 healthy individuals approximately 32 years of age Intervention with a commercial probiotic drink for 4 weeks	No reduction of the *Candida* CFU, reduction of the halitosis score, did not detect *L. casei* after tests
Ishikawa et al. (2015)	*L. rhamnosus, L. acidophilus*, *B. bifidum*	Double-blinded randomized trial (tested group, *n* = 30, control group, *n* = 29) Intervention with trial probiotic products for 5 weeks	Reduction of the *Candida* carrying rate in the tested group
Kraft-Bodi et al. (2015)	*L. reuteri*	Double-blinded placebo RCT, elderly individuals living in a nursing home (tested group, *n* = 84, control group, *n* = 90) Intervention with probiotic lozenges	Improved the *Candida* score
**Animal studies**			
Wagner et al. (1997)	*L. acidophilus*, *L. reuteri*, *L. casei*, *B. animalis*	Oral candidiasis model in immunodeficient bg/bg-nu/nu mice Estimated by the CFU and pathological examinations	Increased the life expectancy in the tested group
Elahi et al. (2005)	*L. acidophilus*, *L. fermentum*	*Candida* infection model using male DBA/2 mice (H-2d), 6–8 weeks of age Oral administration of probiotics	Reduction in the duration of *Candida* colonization in the tested group
Matsubara et al. (2012)	*L. acidophilus*, *L. rhamnosus*	DBA/2 murine oral *Candida* infection model. Control group was treated with nystatin, tested group was treated with probiotics	Reduction of the *Candida* level in the tested group compared with the control group
Zavisic et al. (2012)	*L. plantarum*, *L. casei*	Wister rats and NMRI Ham laboratory mice	Did not show an inhibition in *C. albicans* growth
Ishijima (2012)	*S. salivarius*	ICR mice, oral candidiasis model	Probiotics were not fungicidal, but inhibited *Candida* adhesion
***In vitro* test**			
Chung et al. (1989)	*L. reuteri*	MIC assay using partial purified reuterin	Reuterin, an anti- microbial substance with broad spectrum effects, led to the reduction of *C. albicans growth*
Koll et al. (2008)	*L. plantarum*, *L. paracasei*, *L. salivarius*, *L. rhamnosus*	Antimicrobial activity was detected using the antagonism method	Did not show an inhibition in *C. albicans* growth
Köhler et al. (2012)	*L. rhamnosus*, *L. reuteri*	Antimicrobial activity was detected using an overlay plate or co-culture assay. The genome-wide transcriptional profile of *C. albicans* was assayed with a cDNA microarray	*C. albicans* was antisepticized by inhibition of the metabolic activity under a low pH
Hasslof et al. (2010)	*L. plantarum*, *L. rhamnosus GG*, *L. paracasei*, *L. reuteri*, *L. acidophilus*	Agar overlay interference tests	*Candida* growth was reduced, however, the effect was generally weaker than for mutans streptococci
Jiang et al. (2014)	*L. rhamnosus GG, L. casei*, *L. reuteri*, *L. brevis*, *L. bulgaricus*	Estimated the inhibition effect by pH conditions and the combination of saccharides using EIR	Inhibition capacity differed in the probiotic strains, *L. rhamnosus* showed the strongest inhibition effects against *C. albicans*
Shokryazdan et al. (2014)	*L. acidophilus*, *L. buchneri*, *L. casei*, *L. fermentum*	Co-culture test with 12 pathogenic microorganisms	The active substance was organic acid
Kheradmand et al. (2014)	*L. johnsonii*, *L.plantarum*	After selenium treatment, the antimicrobial effects improved	The active substances were exometabolities or novel anti-*Candida* compounds
Kojima et al. (2015)	*L. fermentum*, *L. plantarum*, *L. paracasei* per 12 species (40 strains)	Co-culture and growth inhibition assays of *C. albicans* with *Lactobacilli* culture supernatant or saccharides	Three saccharides and five strains became candidates for pre- and probiotics, respectively

### *In Vivo* Animal and *In Vitro* Studies of Probiotics for Oral *Candida* Infections

Several *in vivo* animal studies have been performed which have examined the effect of probiotics on oral *Candida* infections. However, the results remain controversial. Some reports suggested a local as well as systemic beneficial effect of probiotics on candidiasis (Wagner et al., 1997; Elahi et al., 2005; Matsubara et al., 2012), while others have not observed a positive effect (Zavisic et al., 2012). These diverse observations may result from differences in the administration technique employed. However, Kojima et al. (2015) demonstrated that the key factor for the effectiveness of probiotics may be the selection of an appropriate strain that works against *Candida.* A diverse set of *Lactobacilli* species has been used for the previous probiotic studies. The genome size of the *Lactobacillus* genus ranges from 1.23–4.91 Mb and the GC content spans 31.9–57.0% among different species (Caufield et al., 2015). In addition, the properties of strains within the same species of *Lactobacillus* have been shown to vary (Koll et al., 2008; Tiihonen et al., 2010). Some of these studies have selected probiotic (*Lactobacillus*) strains that are known to confer intestinal health benefits and presume a similar beneficial effect on oral infections or *Candida* infections. Therefore, it is important to demonstrate the *in vitro* activity of a probiotic strain against *Candida* and subsequently select an efficient strain for *in vivo* and clinical studies. Such studies are few and shown in **Table 1**.

### Anti-*Candida* Products of Probiotics for Oral Candidiasis

Probiotic lactobacilli co-aggregate with *Candida* and produce antimicrobial substances that have a direct growth inhibitory effect on *Candida*. Some of these substances produced include organic acids (e.g., lactic acid and acetic acid), hydrogen peroxide (H_2_O_2_), bacteriocins, and uncharacterized low molecular weight substances with antifungal properties. Lactobacilli universally produce lactic acid that inhibits the metabolic activity of *Candida* sp. (Köhler et al., 2012), which has a weak antifungal activity (Zalán et al., 2010). It appears that lactobacilli do not produce effective concentrations of H_2_O_2_ against fungi (Shokryazdan et al., 2014), unlike other bacteria (Piard and Desmazeaud, 1991).

Lactic acid bacteria produce bacteriocins, proteinaceous antimicrobial substances with molecular weights of several thousand daltons or more. Bacteriocins can be divided into five classes according to their primary structure, molecular composition and properties (Chen and Hoover, 2003; Pascual et al., 2008). Bacteriocin L23 produced by *Lactobacillus fermentum* L23 (Pascual et al., 2008), plantaricin produced by *L. plantarum* (Sharma and Srivastava, 2014), and pentocin TV35b produced by *L. pentosus* (Okkers et al., 1999) appear to be effective against the yeast form of *Candida*. Bacteriocins effective for hyphal forms of *Candida* have not yet been identified (Calderone and Fonzi, 2001; Douglas, 2003). Low molecular substances of lactobacilli, such as reuterin (Talarico et al., 1988), reutericyclin (Ganzle, 2000), and dyacetyl (Jay, 1982), have also been shown to be effective against the yeast forms of *Candida* (Chung et al., 1989).

## Prebiotics

The term “prebiotics” was defined by Gibson and Roberfroid (1995) as “a non-digestible food ingredient that beneficially affects the host by selectively stimulating the growth and/or activity of one or a limited number of bacteria in the colon, and thus improves host health.” Studies of oral prebiotics are limited. Sugars and dietary fiber have been considered to be prebiotics for intestinal lactic acid bacteria (Gibson and Roberfroid, 1995). However, this is not the case for the oral environment, as the presence of sugars increases the risk of dental caries. The mutans group of streptococci metabolizes cariogenic sugars, such as glucose and sucrose, and produces organic acid and insoluble glucan factors that contribute to dental caries. On the other hand, sugar alcohols such as xylitol suppress the growth of *Streptococcus mutans*. Xylitol, a reduced derivative of xylose, converts to xylitol-5-phosphate inside *S. mutans* cells and inhibits glycolysis (Miyasawa-Hori et al., 2006). Similarly, arabinose, a member of the same aldopentose group as xylose, is not assimilated by *S. mutans* (Coykendall, 1977) and likely has a similar effect as xylitol. We recently demonstrated that xylitol, xylose, and arabinose inhibited the growth of *S. mutans*, but were utilized for the growth of most of the lactobacilli strains we tested (Kojima et al., 2015). Although xylitol is generally not assimilated by lactobacilli, a recent report showed that 36% of lactobacilli strains isolated from human oral cavities were able to metabolize xylitol (Almstahl et al., 2013). Meanwhile, our previous data on *Candida albicans* ATCC18804 showed decreased growth in the presence of three saccharides (xylitol, xylose, and arabinose) compared with glucose (Kojima et al., 2015). There are conflicting reports on the ability of *C. albicans* to assimilate xylitol and aldopentose. Mäkinen et al. (1975) and Maleszka and Schneider (1982) showed that *C. albicans* is not capable of proper growth in the presence of xylitol. Uittamo et al. (2011) suggested that xylitol metabolism of *Candida* might compete for the nicotinamide adenine dinucleotide (NADH) coenzyme, leading to the downregulation of alcohol dehydrogenase (ADH). Clinical trials of Turku sugar studies III and VIII showed significantly decreased colony counts and detection frequency of oral *Candida* in the xylitol intake group [Larmas et al. (1974, 1976)]. On the other hand, yeast is known to possess a pentose assimilation pathway that produces ethanol from arabinose and xylose by an enzymatic reaction (Chiang and Knight, 1960; Ônishi and Suzuki, 1966). Even if *Candida* is capable of slowly assimilating those three candidate sugars, the phenomenon of slower growth compared to that of probiotic bacteria may have a competitive inhibition on *Candida*. The presence of xylitol inhibits the adhesion of *Candida* to mucosal surfaces (Pizzo et al., 2000; Abu-Elteen, 2005). In an experimental murine model of gastrointestinal candidiasis, the colonization and invasion of *C. albicans* was significantly reduced in the group supplemented with xylitol compared to the group supplemented with glucose (Vargas et al., 1993).

## Synbiotics

### The Noteworthy Features of Synbiotics Associated with the Oral Application

Gibson and Roberfroid (1995) proposed the use of probiotics and prebiotics fusion products or “synbiotics” for the intestinal tract microbiota (Panigrahi et al., 2008). However, the use of synbiotics for the oral microbiota has not been well studied (Kojima et al., 2015). It is important to understand the limitations associated with the oral application of synbiotics. Probiotic bacteria are not able to easily colonize adult oral cavities (Lazarevic et al., 2010; Tiihonen et al., 2010). Therefore, it appears that synbiotics are more effective for oral applications than probiotics alone. One must, however, consider the risk of dental caries while applying lactic acid bacteria in the oral cavity. Lactobacilli have long been considered to be one of the cariogenic bacteria present in dental plaque (Glass, 1952). Currently, there are two concepts on the association of lactobacilli with dental caries. Lactobacilli comprise a very small proportion of normal oral microbiota and are primarily present on the tongue dorsum, rather than in dental plaque (van Houte et al., 1972). However, they are hardly detected in the oral cavity of caries-free individuals (Yang et al., 2010). The lactobacilli count in the saliva is an indicator of the dental caries activity as lactobacilli penetrate porous tooth surfaces in early caries lesions or adhere to type I collagen exposed in the carious portion of the tooth (Caufield et al., 2015). As the salivary lactobacilli count correlates with the amount and frequency of carbohydrate (sugar) intake (Jay, 1947; Becks, 1950), the presence of lactobacilli is a reliable indicator for the dental caries activity (Crossner, 1981). Therefore, if one can maintain good oral hygiene, oral probiotic therapy with lactobacilli alone may not contribute to the development of dental caries. In addition, if appropriate prebiotics are administered simultaneously, then synbiotic therapy may suppress the development of oral candidiasis.

### The Different Immune Responses Associated with Synbiotics in the Intestinal Tracts or Oral Cavities

The important considerations for synbiotic therapy of the intestine and oral cavity are the host immune component and reactions. While activation of a substantial host immune response can be expected in the intestine, a similar phenomenon is not expected in the oral cavity as it is not an organ of mucosa-associated lymphoid tissues (MALT). In the intestine, probiotic bacteria are incorporated into M cells in Peyer’s patches (PP), which is a major component of gut-associated lymphoid tissues (GALT), and digested to form active antigens. Macrophages and dendritic cells in PP phagocytize probiotic bacteria and are activated to produce several cytokines, which stimulate T-cell and B-cell functions (Matsuzaki et al., 2007). Moreover, daily supplementation of lactobacilli as part of a normal diet increased the number and activity of natural killer cells in healthy elderly individuals (Tiihonen et al., 2010). Thus, synbiotics in the intestinal tract can be expected to activate both innate immunity and acquired immunity of cell-mediated and humoral immunity. Conversely, the oral cavity it is not an immune organ and phenomenon such as direct antigen presentation to adaptive immune cells does not occur. Nevertheless, some probiotic clinical trials and animal studies using oral candidiasis models have reported an increase of sIgA against *Candida*, leading to the suppression of *Candida* in the oral cavity (Wagner et al., 1997; Elahi et al., 2005; Mendonça et al., 2012). It is well known that secretion of sIgA at the salivary gland is through differentiated plasma cells from B cells stimulated at MALT. According to the results of clinical and animal studies described above, oral synbiotics appear to transition into intestinal synbiotics, as the oral cavity is connected to the intestine. Children who were oral lactobacilli carriers were found to have similar lactobacilli in their feces (Caufield et al., 2015). Hence, it appears that the intestinal colonization of lactobacilli is transmitted though the oral cavity, which may provide simultaneous synbiotic activity at the oral cavity and the intestine.

## Biogenics

Previous studies have highlighted the limitation of colonization and fixation of non-nature probiotic bacteria in the intestinal tracts of human bodies (Haenel, 1960; Mitsuoka and Kaneuchi, 1977). This scenario is also relevant for the probiotic application in oral cavities, particularly when considering the associated risk of oral probiotics and dental caries. In order to address foregoing concerns, the concept of “biogenics” has been suggested as a solution (Mitsuoka, 2000). Biogenics is defined as “food ingredients which beneficially affect the host by directly immunostimulating or suppressing mutagenesis, tumorigenesis, peroxidation, hypercholesterolemia, or intestinal putrefaction” (Mitsuoka, 2000). Hence, previous studies have suggested the administration of non-viable probiotic bacteria to obtain some “probiotic” effects. It was reported that the consumption of pasteurized fermented milk elongated the lifespan of mice (Arai et al., 1980; Takano et al., 1985). A significant reduction of the Ehrlich ascites tumor growth in mice was also reported (Takano et al., 1985). In addition, it was shown that heat-inactivated *Enterococcus faecalis* (Terada et al., 2004) or *L. gasseri* (Sawada et al., 2016) retained a beneficial regulatory function in the gut. Moreover, Nakamura et al. (1995) identified an angiotensin I-converting enzyme (ACE) inhibitor in a Japanese sterilized milk beverage fermented by *L. helveticus* and *Saccharomyces cerevisiae.* The active substance was lactotripeptides metabolically generated in the fermentation pathway. Follow-up studies were able to determine the bioactive metabolites of probiotic bacteria in addition to the antimicrobial substances, such as bacteriocin (Ross et al., 2010; O’Shea et al., 2012), and other beneficial active substances, such as conjugated linoleic acid (CLA; Hayes et al., 2006; Ross et al., 2010; O’Shea et al., 2012), protein or peptides (Möller et al., 2008; Bogsan et al., 2013), and polyphenols (Dharmaraj, 2010; Monagas et al., 2010). Taking all these observations into account, the new concept, biogenics, which makes use of the bioactive metabolites as foods or medicine, was recently advocated (Mitsuoka, 2000, 2014). The biogenics effect is independent of the colonization and viability of probiotic bacteria. Hence, biogenics is the direct delivery of an isolated and purified active ingredient of probiotics to the local environment. This strategy may also be used as an antifungal therapy. It may be possible to purify the active ingredients of probiotic bacteria that demonstrate antifungal activity for use in the biogenics process. However, this idea requires further study before clinical use.

## Conclusion

**FIGURE 1:**
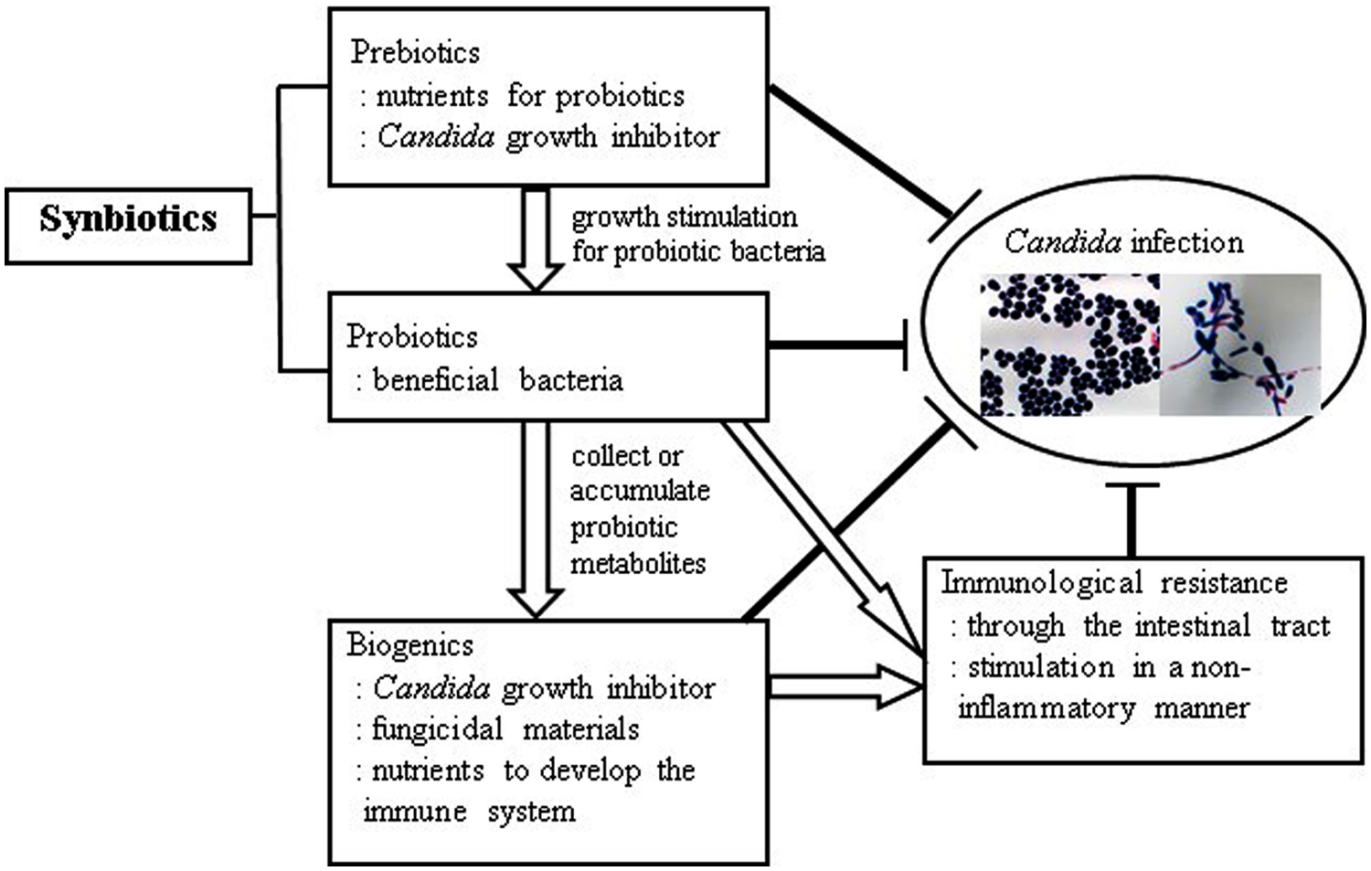
**Anti-*Candida* effects with synchronized prebiotics, probiotics, biogenics, and immunological resistance**.

Taking the abovementioned studies into consideration, it is conceivable that an innovative combination of prebiotics, probiotics, synbiotics, and biogenics instrumental in devising a successful, novel antifungal regime in the future (**Figure 1**). More comprehensive investigations on the mechanism of synbiotics and biogenics are needed for this purpose. Hence, more studies are warranted to examine the bioactive metabolites of probiotic bacteria that induce favorable immunological outcomes and suppress *Candida* infection in the human oral cavity.

## Author Contributions

TO made the description plan of this review article, and carried out the manuscript writing and figure charting. YK, CS, and NM read carefully and made arrangements on the manuscript according their discussions.

## Conflict of Interest Statement

The authors declare that the research was conducted in the absence of any commercial or financial relationships that could be construed as a potential conflict of interest.
